# Effect of ionizing radiation on the proliferation of human embryonic stem cells

**DOI:** 10.1038/srep43995

**Published:** 2017-03-07

**Authors:** Irina V. Panyutin, Sonia A. Holar, Ronald D. Neumann, Igor G. Panyutin

**Affiliations:** 1 Department of Radiology and Imaging Sciences, Clinical Center, National Institutes of Health, Bethesda, MD 20892, USA; 2 Department of Chemical Engineering, Massachusetts Institute of Technology, Cambridge, MA 02139, USA.

## Abstract

We studied the effect of ionizing radiation (IR) on continuous growth of seven hESC lines. Cells were exposed to 0, 0.2, or 1 Gy of X-rays, and the growth rates of cell populations were assessed by measuring areas of the same individual colonies versus time. The population doubling times (DT) of sham-irradiated cells varied from 18.9 to 28.7 hours for different cell lines. All cell lines showed similar reaction to IR, i.e. cell populations dropped within 24–48 hours post IR; after that they recovered and grew with the same rate as the sham-irradiated cells. The relative cell survival (RCS), i.e. the ratio of normalized cell population in the irradiated samples to that of the sham-irradiated ones varied from 0.6 to 0.8 after 0.2 Gy, and from 0.1 to 0.2 after 1 Gy IR for different cell lines. We found that the RCS values of hESC lines correlated directly with their DT, i.e. the faster cells grow the more radiosensitive they are. We also found that DT and RCS values of individual colonies varied significantly within all hESC lines. We believe that the method developed herein can be useful for assessing other cytotoxic insults on cultures of hESC.

Human embryonic stem cells (hESC) are unique models for studying genotoxic stresses including those produced by ionizing radiation (IR) because of their virtually unlimited proliferation potential^1^,^2^. In addition, their ability to differentiate into various tissues allows for studying the effects of IR on the developmental processes^3^. Studies of the effects of IR on hESC are also important because of the prospects of using these cells in regenerative medicine, which would require their transplantation or the transplantation of their differentiated products, and the imaging of the transplants with various techniques that may, like positron emission tomography (PET), utilize ionizing radiation^4^.

During the past decade considerable knowledge was gained regarding the effects of IR on hESC^5^. It was shown that hESC are very sensitive to IR; the exposure to a relatively low dose of 1 Gy results in death of almost 30% of the cells^6^. The immediate effect of IR exposure of these cells is apoptosis; however, the surviving cells retain their pluripotency markers and ability to form all three embryonic germ layers as was evidenced by teratoma formation assay^6^,^7^,^8^. At the same time hESC have enhanced capacities to repair DNA damage as compared to differentiated cells^9^. It was found that these cells lack the G1/S checkpoint and stop their cell cycle progression after IR exposure by G2/M arrest instead^10^,^11^,^12^. Repair of DNA double strand breaks in hESC was shown to rely largely on homologous recombination^13^,^14^,^15^.

A distinct feature of hESC is that in culture they propagate in tightly associated colonies^16^. When dissociated to a single cell suspension and plated back to a culture dish the majority of the cells die. Although treatments with certain factors can increase the plating efficiency of hESC^17^,^18^, proliferation in a colony is a normal physiological state of these cells^19^,^20^,^21^. It was also shown that colony morphology is closely correlated with the maintenance of hESC pluripotency^22^.

The hallmark of IR exposure of cells in culture is the loss of their ability to proliferate, which happens within several cell divisions upon the exposure. Therefore, the classical assay to measure the effect of IR on cells in culture is the clonogenic survival assay that measures proportion of the cells that retain the ability to proliferate (to form new colonies) after the exposure^23^. However, the colony-based growth of hESC makes it impossible to apply the classical clonogenic survival assay to study the effect of IR on these cells.

Another technique to assess the cytotoxicity is to measure the growth rate of cells in culture^23^. The classical way to measure cell growth is to seed the equal amounts of cells into control and experimental dishes, and count the number of surviving cells with time by counting live cells or by measuring the expression of proliferation markers. These approaches also could not be directly applied to hESC because their colonies are usually heterogeneous in size and breakage of the colonies to a single cell suspension for accurate counting will result in massive cell death. Therefore, there is no simple way to disperse hESC in equal amounts to multiple wells.

Herein, we propose to measure growth curves of hESC to assess the effects of IR on their proliferation by continuously measuring the areas of their colonies. Previously we applied this method to assess the effect of radioiodine treatment on hESC^24^. Here we extended these studies to measure the effect of X-rays on seven commonly used hESC lines.

## Results

### Apoptotic Death of hESC after exposure to IR

**Figure 1:**
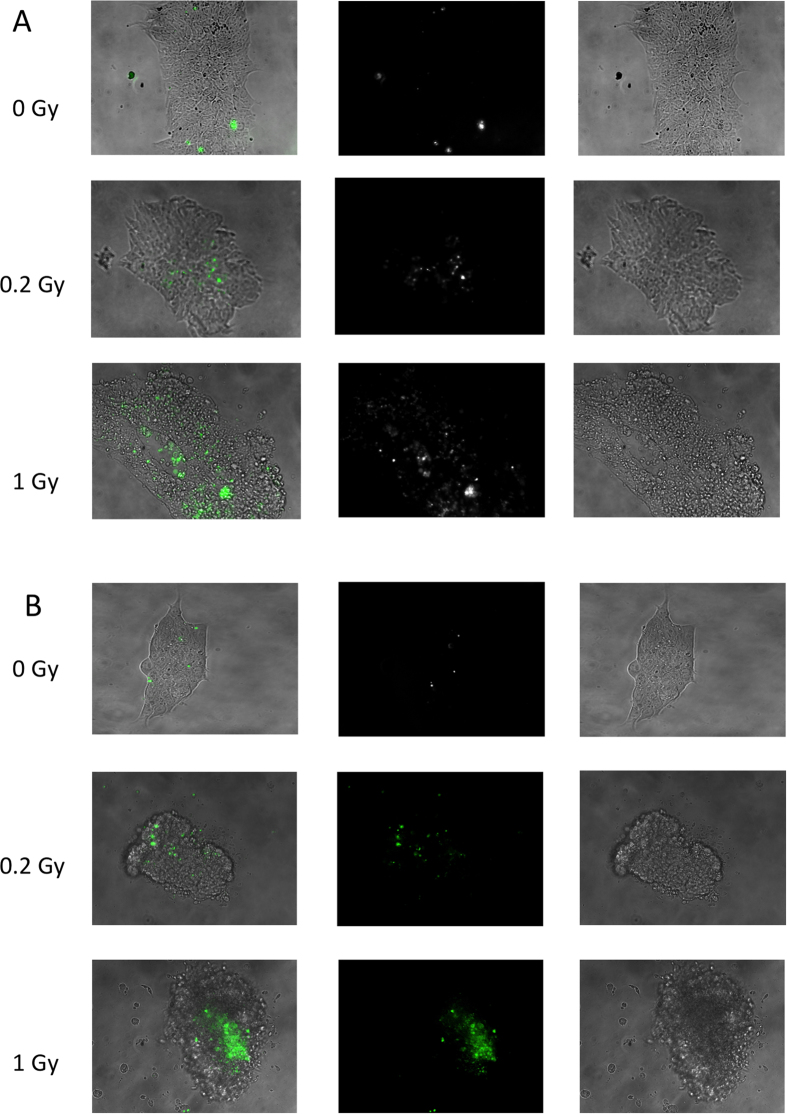
Apoptosis in hESC after exposure to IR. (**A**) H1 and (**B**) H9 hESC were exposed to 0, 0.2, or 1 Gy IR, and after 3 hours were stained for Caspase-3/7 activity (green fluorescence signal). First column is combined bright field and fluorescent images, second column is the fluorescent image, and third column the bright field image alone.

It was documented that IR exposure results in fast apoptosis of hESC^6^,^7^,^11^,^12^. To confirm these observations in our cell culture conditions we used a fluorescent peptide-based assay to detect activated Caspase 3/7. The results of these assays for two hESC lines, H1 and H9 are shown in Fig. 1. Other cell lines showed similar results (data not shown). In the sham-irradiated colony there are only few cells that exhibit fluorescence. However, 3 hours after irradiation with 0.2 Gy there are numerous fluorescence-positive cells that undergo apoptosis, and the number of apoptotic cells is considerably higher after 1 Gy irradiation (Fig. 1). In addition, the edges of the colonies of IR-exposed cells are not smooth reflecting the blebbing characteristic of the cells that undergo apoptosis. Detached from the colonies free-floating cells are also observed, especially after 1 Gy of irradiation. Interestingly, these cells are not positive for the activated Caspase 3/7 staining. This could indicate only temporal activity of Caspase 3/7 during apoptosis, or possibly another pathway of cell death after exposure to IR^25^.

### Growth Curves of hESC lines after exposure to 0, 0.2 and 1 Gy IR

**Figure 2:**
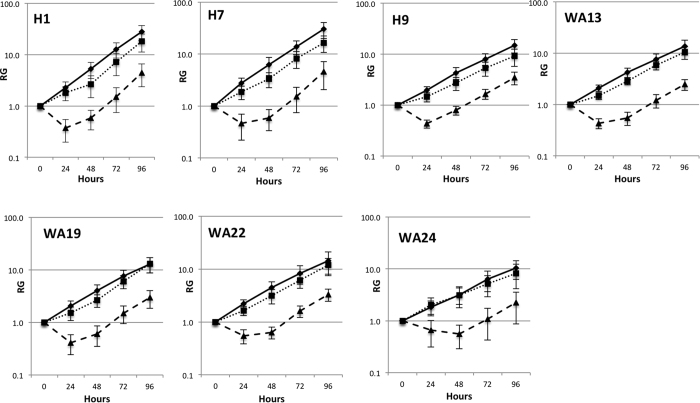
Growth curves of seven hESC lines exposed to 0 Gy (solid lines), 0.2 Gy (dotted lines), and 1 Gy (dashed lines) of IR. Relative growth (RG) values were calculated as ratios of the area of the colonies at various time points to their areas before IR (0 hours). Each point represents an average of RG measurement for at least 20 colonies. Error bars show STD.

**Figure 3:**
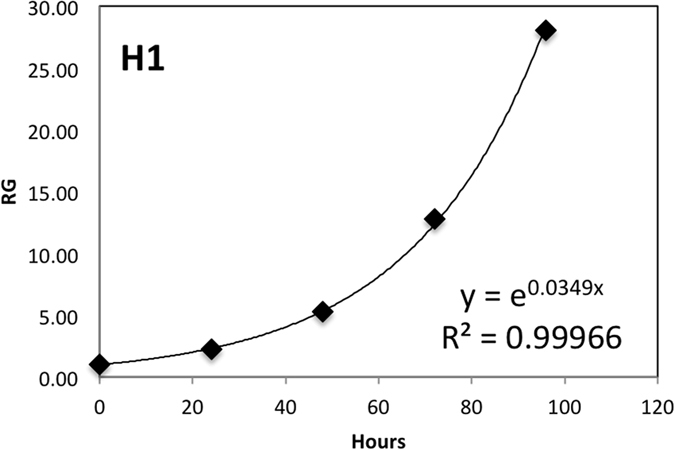
Growth curve of H1 hESC not exposed to IR. Relative growth (RG) values were calculated as ratios of the area of the colonies at various time points to their areas before IR (0 hours). Each point represents average of RG measurement for 20 colonies. Experimental points were fitted by equation **y = y**_**0**_** • e**^**αt**^ and then DT was calculated using formula **DT = 1/(α • log**_**2**_**(e)).**

Seven lines of hESC were seeded in 6-well plates, and after 24 hours were irradiated with 0 Gy (sham irradiation), 0.2 Gy, or 1.0 Gy of X-rays as described in the methods section. Exposure to doses of IR higher than 1 Gy resulted in significant cell death, while doses lower than 0.2 Gy resulted in a negligible effect on cell growth. Immediately after exposure to IR the cells were placed in the CO_2_ incubator. The area of the colonies was measured immediately before irradiation (0 hours time point) and again upon 24 hour intervals up to 96 hours. We defined relative growth (RG) as the ratio of the area of the colonies at various time points to that at 0 hours. The resulting growth curves are shown in Fig. 2. The sham-irradiated cells grew exponentially; i.e., their growth curves are close to linear in the logarithmic scale (Fig. 2). We measured growth rates and doubling times for the hESC lines by fitting data points with exponential equations as described in the methods section. An example of such a fit for H1 hESC lines is shown in Fig. 3. The growth rates and doubling times for all cell lines are summarized in Table 1. The obtained doubling times are in agreement with previous measurements^26^,^27^, and vary considerably between hESC lines; H7 and H1 showed the fastest growth, while WA24 was the slowest.

The cell colonies exposed to 0.2 Gy of IR show only a slight delay in their growth (Fig. 2). The colonies of the cells irradiated with 1 Gy IR showed significant reduction in their area reflecting substantial cell death. However, after 24 to 48 hours the cell growth recovered to the original rate; thus, the growth curves of the irradiated cell colonies are almost parallel to those of the sham-irradiated cells on a logarithmic scale (Fig. 2).

### Effect of growth rate on the radiosensitivity of hESC lines

For characterization of the reduction in cell populations and the delay in proliferation of hESC after IR exposure we use a relative cell survival (RCS) index defined as the ratio of the relative growth of the colonies of the IR exposed cells to that of the sham-irradiated cells at different times post-irradiation.

**Figure 4:**
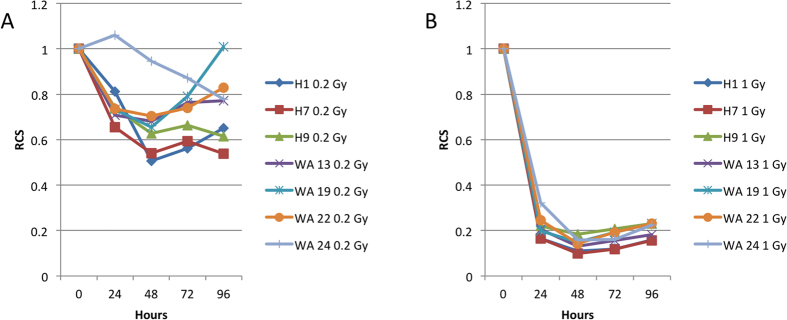
Relative Cell Survival (RCS) of seven hESC lines after (**A**) 0.2 G and (**B**) 1 Gy IR. RCS was calculated by division of average RG of 0.2 or 1 Gy colonies to average RG of 0 Gy colonies.

These RCS values at different time points for different cell lines after exposure to 0.2 Gy and 1.0 Gy IR are shown in Fig. 4A and B correspondingly. The RCS values after 0.2 Gy IR were in general between 0.5 and 1.0. WA24 cells showed higher RCS values at earlier time points which reflects their delayed reaction to IR. Exposure to 1.0 Gy IR resulted in RCS between 0.1 and 0.2. Again, the slowest growing WA24 cell line showed the highest RCS.

**Figure 5:**
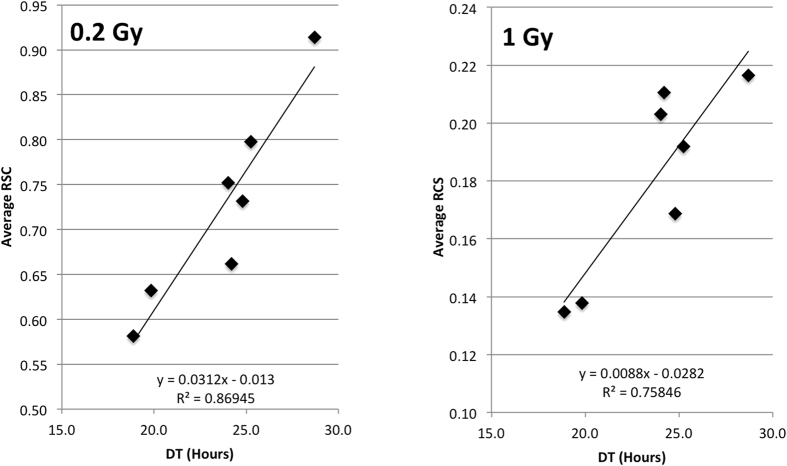
Correlation plots of average RCS vs DT for seven hESC lines.

To test if there is an association between RCS and growth rate of hESC lines we plotted RCS values averaged over all time points (except 0 hours) versus doubling times (Fig. 5). The results show clear correlation between RCS and doubling time for hESC lines at both 0.2 Gy and 1.0 Gy IR dose, with correlation coefficients R^2^ = 0.87 and R^2^ = 0.76 respectively. In other words we found that the slower the growth rate of a hESC line the more cells survive IR exposures.

### Variability of growth rate between colonies of hESC

**Figure 6:**
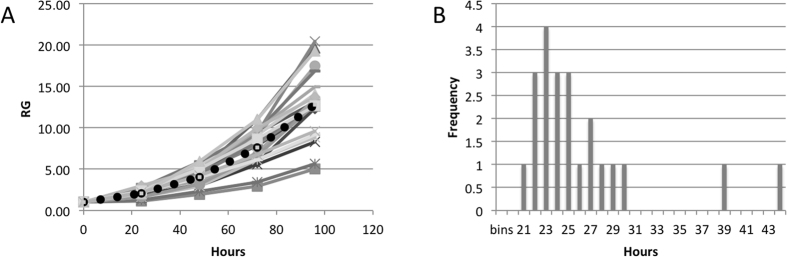
Variability in growth rates between colonies. (**A**) Growth curves of 23 colonies of WA19 hESC line; black dots show the average growth curve. (**B**) Frequency distribution of DT for 23 colonies of WA19 hESC line.

The above results describe average values for at least 20 colonies. We also noticed a significant variability in the growth rate between individual colonies of hESC lines, and decided to further investigate this phenomenon. Figure 6A shows growth curves for 23 colonies of WA19 hESC. Each of these growth curves was fitted with an exponential function to determine growth rate and doubling times of individual colonies. The distribution of the doubling times for the colonies is shown in Fig. 6B. Most of the colonies have DT between 20 and 30 hours, however there were two outliers with 39 and 44 hours DT; the average DT for this cell line was 25.3 hours (Table 1).

**Figure 7:**
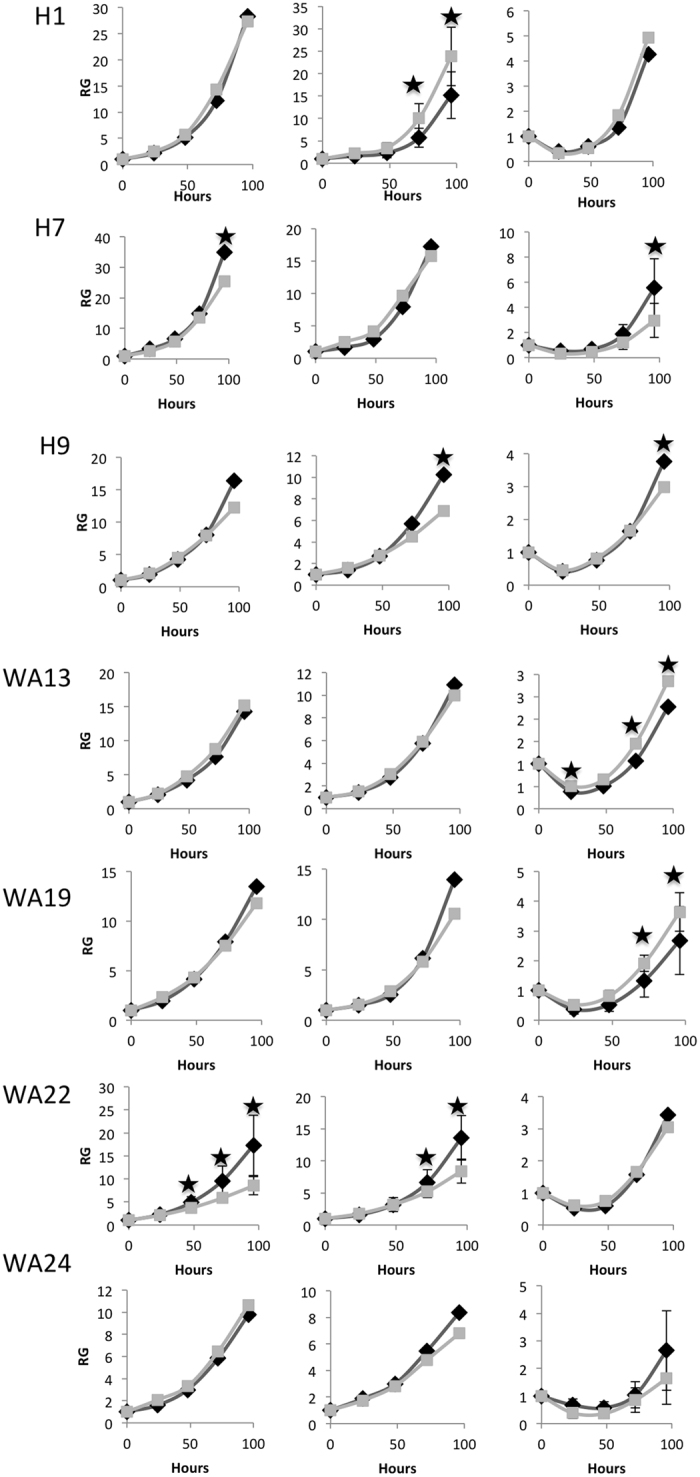
Growth curves of colonies with sizes above (gray lines) and below (black lines) of mean size values for seven hESC lines. Stars show time points where the differences between the larger and smaller colonies were statistically significant (p < 0.05).

All studied hESC lines show significant variation in growth rate between individual colonies. Whiskers plots shown in Supplementary Figure 1 summarize the variability in RCS between individual colonies at 96 hours time point. There were also considerable differences in the sizes of individual colonies. Graphs shown in Supplementary Figure 2 show the variations in the colony sizes for all seven hESC lines. The average sizes of the colonies for hESC lines under investigation are shown in Supplementary Table 2. We did not find any correlation between the average colony sizes and average RCS values for different cell lines. To further investigate this issue we compared growth curves for colonies with sizes above and below mean size value. The results of such analyses are shown in Fig. 7(A and B). In the sham irradiated samples (0 Gy) small colonies tended to grow faster than the larger ones. For H7 and H9 cell lines that finding could be explained by the fact that at the later time points the larger colonies became overgrown and cells start “piling up”. However, for WA22 hESC the smaller colonies grew faster than the larger ones from the earliest time points; this is also true after 0.2 Gy IR for this cell line. There are several examples in which, after exposure to IR the larger colonies grow faster than the smaller ones; i.e. H1, 0.2 Gy; WA13 and WA 19, 1 Gy. However in other cases, as with the H7 and H9 cell lines the smaller colonies grew faster than the larger colonies. Therefore, we could not establish a common correlation between the growth rate and the size of colonies for the studied hESC lines.

## Discussion

Using an increase in the colony area as a surrogate measurement for cell proliferation, we assessed the effect of IR on seven lines of hESC. We confirmed the dose-dependent increase in apoptosis immediately after exposure of hESC to IR. However, we cannot exclude that other pathways of cellular death are involved as this has been observed in the past^25^.

After an initial drop in the cell population all cell lines show recovery of their growth after 24 to 48 hours. We use RCS, i.e. the ratio of RG of irradiated cells to RG of sham-irradiated cells as a measure of their radiosensitivity. We found that RCS varied between different hESC lines. The RCS of the most sensitive H7 cell line was ca. 1.5 times lower than that of the least sensitive WA24 cells. Interestingly, we found strong direct correlation between RCS and cell population DT meaning that faster growing hESC are more prone to cell death than slower growing cell lines after exposure to IR. One possible explanation of this phenomenon could be that the faster growing cell lines have a higher percentage of cells in S phase. It was shown^11^ that exposure to IR results in an increased percentage of hESC in G2 phase and a depletion of S-phase cell population. That could be also interpreted as a higher sensitivity to IR of those cells in S phase that cannot effectively repair their damaged DNA during replication and thus predominantly die from apoptosis.

Remarkably, we found a significant difference in DT and RCS between individual colonies of the same cell line that is comparable or even larger than the difference in these parameters between different hESC lines. We could not establish a clear correlation between RCS and the size of the colony. We believe that DT and RCS could be characteristic features of individual colonies that are set during initial colony establishment from single cells. In hESC colonies cells are tightly packed and interconnected^21^. Organization in colonies is important for maintenance of pluripotency of hESC cultures^28^. It is possible that the colony controls proliferation, prevents differentiation and controls competition of individual cells perhaps via p53-based mechanism like that which was shown for the mouse iPSC cultures^29^. In other words cells are interdependent within the colony and the survival of the colony is more important than the survival of individual cells; i.e. the colony is the true unit of the hESC population^21^. Therefore, dissociation of a colony to a single cell suspension, which is commonly used in the literature to assess hESC growth rate^30^,^31^ may result in a different estimation of DT, at least due to the lag period needed for re-establishing the colony. Certainly more studies are required to test this hypothesis.

Cultures of hESC have become popular models in toxicology studies. They are also widely used in studies regarding the roles and functions of important genes in embryonic development. The method presented herein is simple, sensitive, and can be easily automated. It also assesses cultures of hESC in the most physiologically relative format, i.e. growing in colonies. We believe that our approach will be useful in studies on the effects of various treatments and changes in gene expression on the proliferation of hESC.

## Methods

Human Embryonic Stem cell lines: WA01 (H1), WA07 (H7), WA09 (H9), WA13 (H13), WA19, WA22, and WA24 (WiCell Research Institute, Madison, WI, USA) were maintained in feeder-independent cell culture environments for human embryonic stem cells on BD Matrigel hESC-qualified Matrix (BD Biosciences, San Jose, CA, USA) coated BD Falcon^TM^ qualified surfaces plated in serum-free defined mTeSR1 media (Stem Cell Technology Inc, Vancouver, Canada) at 37 °C and 5% CO_2_. The characteristics of the hESC cell lines used in this study are summarized in Supplementary Table 1. Growing cells were passaged using collagenase IV. Media was changed every day. For experiments cells were transferred to 6-well plates and grown for 2 days before irradiation. Then cells were exposed to 0.2 or 1 Gy using Precision X-Ray X-Rad 320, operating at 300 kV/10 mA with a 2 mm aluminium filter with a dose rate of 2.42 Gy/min (242 rad/min) and the control cell cultures were treated with sham-radiation. Cultures of irradiated hESC were maintained at 37 °C and 5% CO_2_ at the conditions described above.

Areas of colonies were measured at the indicated time points starting from the 0 time point taken right before the IR as described in ref. 24. Briefly, hESC were grown on gridded plates to help locate individual colonies at various time points. Phase-contrast images of the cultures were produced by Axiovert 200 microscoipe (Carl Zeiss, Thornwood, NY, USA). Care was taken to minimize time that live cultured cells spend under the microscope; flasks were immediately placed back into the CO_2_ incubator after imaging. The area of the colonies was measured with AxioVision Software (Carl Zeiss) by manually outlining the borders of a colony.

We previously validated the use of colony area as a surrogate marker by direct measurement of cell density within colonies^24^. There was significant variation in cell density between individual colonies, however we found no correlation between the size of the colonies and the cell density within them. Therefore we concluded that the size of a colony as it grows is proportional to the number of cells within it^24^.

At least 20 individual colonies were measured for each time point. Average, mean, standard deviation, and Student’s t-Test p values were calculated using Microsoft Excel. We defined relative growth values (RG) as ratios of the area of the colonies at various time points to their areas before exposure IR. Thus measured RG values were fitted by equation **y = y**_**0**_** • e**^**αx**^ where **α** is the growth rate, and then DT was calculated using formula **DT = 1/**(**α • log**_**2**_(**e**)).

For detection of Caspase-3/7 activity hESC were grown for 2 days on tissue culture treated glass slides (BD Falcon, Bedford, MA), and were exposed to 0, 0.2, or 1 Gy using Precision X-Ray X-Rad 320 irradiator. After irradiation cells were incubated for 3 hours in a cell culture incubator and then labelled with CellEvent™ caspase-3/7 green detection reagent (Invitrogen, Carlsbad, CA, USA) at a final concentration of 5 μM in the culture medium. Cell were incubated for 30 min at 37 °C in the dark. Stained cells were observed with the Zeiss Axiovert 200 fluorescence microscope^6^.

## Additional Information

**How to cite this article:** Panyutin, I. V. *et al*. Effect of ionizing radiation on the proliferation of human embryonic stem cells. *Sci. Rep.* **7**, 43995; doi: 10.1038/srep43995 (2017).

**Publisher's note:** Springer Nature remains neutral with regard to jurisdictional claims in published maps and institutional affiliations.

## Supplementary Material

Supplementary Materials

## Figures and Tables

**Table 1:** Growth rates and doubling times of hESC lines.

	Growth rate (α, 1/hour)	Doubling time (hours)
H1	0.035	19.8
H7	0.037	18.9
H9	0.029	24.2
WA13	0.028	24.8
WA19	0.028	25.3
WA22	0.029	24.0
WA24	0.024	28.7
