# Supplementary material for: Effect of ionizing radiation on the proliferation of human embryonic stem cells
Source: Sci Rep. 2017 Mar 7;7:43995. doi: 10.1038/srep43995 (PMC5339810; doi:10.1038/srep43995)
Supplement: Supplementary Materials [file srep43995-s1.pdf]

Supplementary Materials

Effect of ionizing radiation on the proliferation of human embryonic stem cells.

Irina V. Panyutin, Sonia A. Holar, Ronald D. Neumann, Igor G. Panyutin

Supplementary Figure Legends.

Supplementary Figure 1. Variations in RCS at 96 hours post-IR between individual colonies of seven hESC lines. The horizontal lines in the boxes show first quartile, median, third quartile, while the vertical lines extend to the minimum and the maximum values. For comparison all plots are normalized such that third quartile corresponds to 100%.

Supplementary Figure 2. Variations in colony areas at 96 hours post-IR between individual colonies of seven hESC lines. The horizontal lines in the boxes show first quartile, median, third quartile, while the vertical lines extend to the minimum and the maximum values. For comparison all plots are normalized such that third quartile corresponds to 100%.

Supplementary Table 1. Characteristics of the hESC lines used in this study.

hESC line	Karyotype	Blood type	WiCell stock passage	Experimental passage
WA 01 (H1)	46, XY	0 Rh+	21	22
WA 07 (H7)	46, XX	B Rh+	31	32
WA 09 (H9)	46, XX	A Rh+	24	26
WA 13 (H13)	46, XY	B Rh+	31	32
WA 19	46, XY	A Rh+	9	10
WA 22	46, XX	0 Rh+	12	13
WA 24	46, XY	A Rh+	12	13

Supplementary Table 2 Average colony size (μm^2)

	**H1**	**WA24**	**H7**	**WA19**	**H9**	**WA13**	**WA22**
**0Gy**	55083	55839	50939	146027	104516	61814	201286
**0.2Gy**	39677	36034	155824	117587	142865	180730	132875
**1.0Gy**	73761	83873	154967	461352	411727	379633	367132

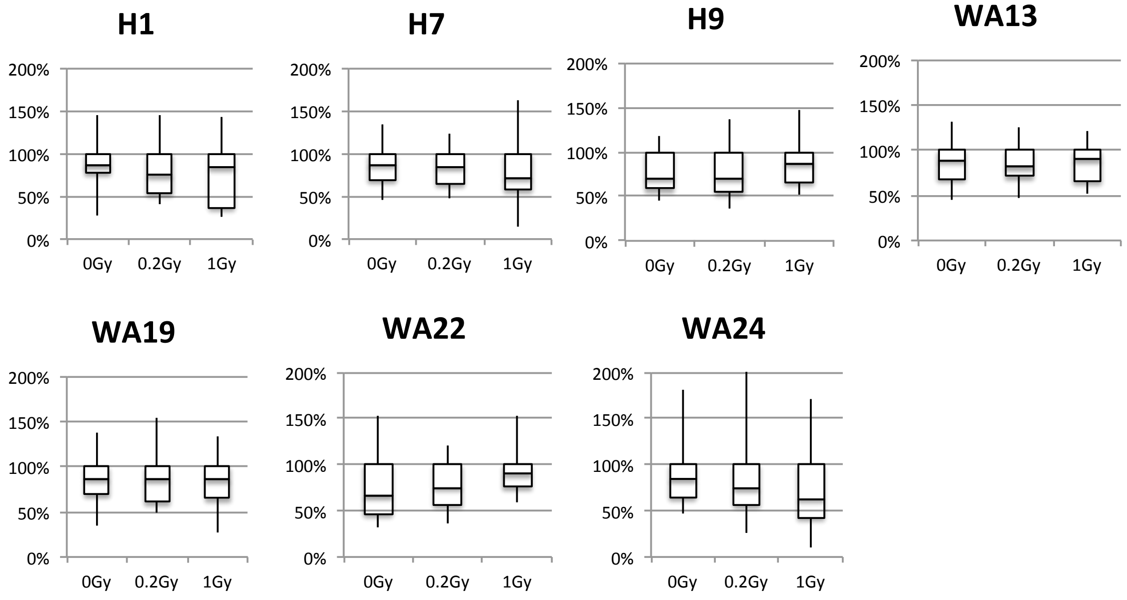

Supplementary Figure 1

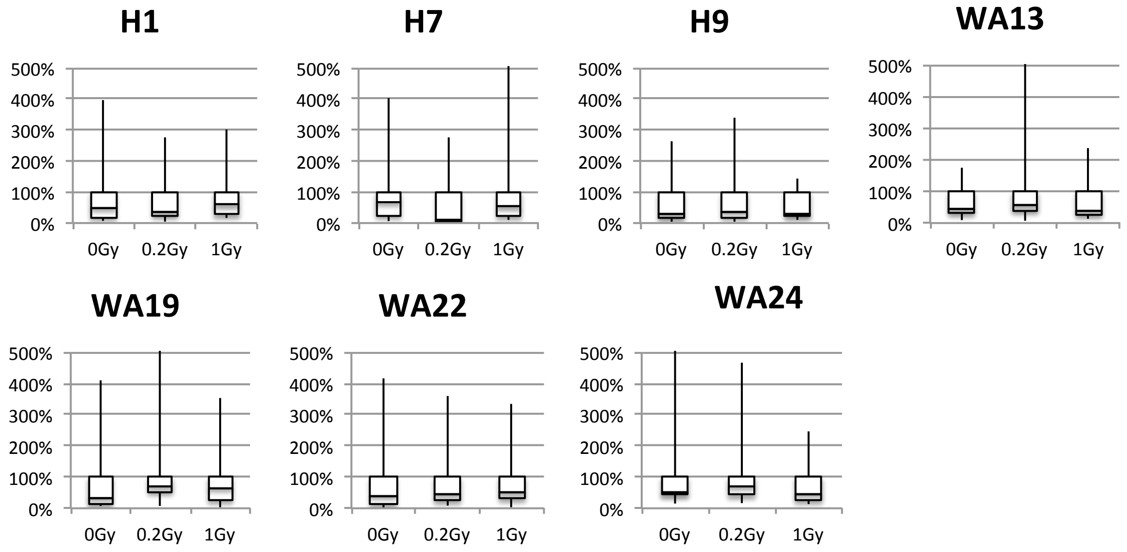

Supplementary Figure 2
